# Article title: Transcriptional profiling efficacy to define biological activity similarity for cosmetic ingredients’ safety assessment based on next-generation read-across

**DOI:** 10.3389/ftox.2022.1082222

**Published:** 2022-12-21

**Authors:** Jorge M. Naciff, Yuquing K. Shan, Xiaohong Wang, George P. Daston

**Affiliations:** The Procter and Gamble Company, Mason, OH, United States

**Keywords:** biological activity, read-across, parabens, methylxanthines, transcriptional profiling

## Abstract

The objective of this work was to use transcriptional profiling to assess the biological activity of structurally related chemicals to define their biological similarity and with that, substantiate the validity of a read-across approach usable in risk assessment. Two case studies are presented, one with 4 short alkyl chain parabens: methyl (MP), ethyl (EP), butyl (BP), and propylparaben (PP), as well as their main metabolite, p-hydroxybenzoic acid (pHBA) with the assumption that propylparaben was the target chemical; and a second one with caffeine and its main metabolites theophylline, theobromine and paraxanthine where CA was the target chemical. The comprehensive transcriptional response of MCF7, HepG2, A549 and ICell cardiomyocytes was evaluated (TempO-Seq) after exposure to vehicle-control, each paraben or pHBA, CA or its metabolites, at 3 non-cytotoxic concentrations, for 6 h. Differentially expressed genes (FDR ≥0.05, and fold change ±1.2≥) were identified for each chemical, at each concentration, and used to determine similarities. Each of the chemicals is able to elicit changes in the expression of a number of genes, as compared to controls. Importantly, the transcriptional profile elicited by each of the parabens shares a high degree of similarity across the group. The highest number of genes commonly affected was between butylparaben and PP. The transcriptional profile of the parabens is similar to the one elicited by estrogen receptor agonists, with BP being the closest structural and biological analogue for PP. In the CA case, the transcriptional profile elicited of all four methylxanthines had a high degree of similarity across the cell types, with CA and theophylline being the most active. The most robust response was obtained in the cardiomyocytes with the highest transcriptional profile similarity between CA and TP. The transcriptional profile of the methylxanthines is similar to the one elicited by inhibitors of phosphatidylinositol 3-kinase as well as other kinase inhibitors. Overall, our results support the approach of incorporating transcriptional profiling in well-designed *in vitro* tests as one robust stream of data to support biological similarity driven read-across procedures and strengthening the traditional structure-based approaches useful in risk assessment.

## Introduction

Assuring safety of cosmetic ingredients has become more challenging given the ban on animal testing for cosmetic ingredients that has been in place since 2013 (Cosmetics Directive 76/768/EEC). To address these issues, Alexander-White et al. (2022) recently described a framework to perform a next-generation risk assessment (NGRA) based on a read-across (RAX) using chemical properties, *in silico* toxicology predictions and integrating data derived from new approach methodologies (NAM) to better substantiate biological activity similarity, and thus similarity in potential toxicity of chemicals being used in a RAX assessment. Two cases studies describing the application of this framework have been published to date, with the first using parabens (Ouedraogo et al., 2022) and with a second using caffeine (Bury et al., 2021). New approach methods (NAMs) data includes transcriptional profiling data from exposed cultured cells, as one of the streams of data to inform about chemical specific biological activity, and this work addresses the use of this stream of NAM data. The basic tenet of RAX is the extrapolation of the outcome of a specific toxic (or lack thereof) *in vivo* endpoint from a tested chemical (source) to a similar (target) chemical. A robust RAX assessment requires the demonstration of similarity not just in physicochemical properties, reactivity and metabolism (Wu et al., 2010) between the chemicals used for the RAX, but also in their biological activity.

Gathering information to define the biological activity of both the source and the target chemical is critical to determine whether the two chemicals act *via* the same mode of action and thus pose a similar hazard *in vivo*. Changes in gene expression are part of an integrated physiological response to chemical exposure of a living organism and these changes represent the response to the biological activity of this chemical on a target organ, which could end in an adverse outcome depending on the concentration and time of exposure. Identifying gene expression changes elicited by a specific chemical exposure, allows the identification of molecular events and cellular pathways affected by such an exposure that could lead to an adverse outcome (De Abrew et al., 2016; Lichtenstein et al., 2020; Chen et al., 2021). Part of this transcriptional response can be also exhibited by cultured cells, representative of target organs (Naciff et al., 2016; De Abrew et al., 2019; Alarcan et al., 2022) and this information offers the opportunity to determine the biological activity associated with a particular chemical, and with that define its mode of action. Comparison of the gene expression changes elicited by structurally related chemicals will substantiate the biological basis for RAX. We (De Abrew et al., 2015; De Abrew et al., 2016; Naciff et al., 2016; De Abrew et al., 2019) and others (Dreser et al., 2015; Rempel et al., 2015; Yeakley et al., 2017; Peshdary et al., 2021; Escher et al., 2022) have started trials in this direction with encouraging results. For example, we (De Abrew et al., 2019) have used transcriptional profiling to identify biologically similar chemicals for m-ethyl phenol and 4-chloro-1,3-diaminobenzene, identifying m-cresol as the closest biological analogue of m-ethyl phenol; while 4-chloro-2-methylaniline hydrochloride and 2-chloro-1,4-diaminobenzene sulfate as the closest biological analogues of 4-chloro-1,3-diaminobenzene. Peshdary et al. (2021) used transcriptional profiling to explore similarities and differences between bisphenol A (BPA) and three of its analogues, bisphenol S (BPS), bisphenol F (BPF) and 3,3′,5,5′-tetrabromobisphenol A in the human embryonic stem cell line H9 (WA09). Peshdary et al. determined that BPA, BPF, and BPS have similar potencies in inducing transcriptional changes and perturb many of the same pathways, while TBBPA was the least structurally similar bisphenol of the group and had much lower potency. Escher et al. (2022) used transcriptional profiling to determine biological similarity between thirteen structurally similar branched aliphatic carboxylic acids and determined that the closest analogues shared the most similar transcriptional profile in HepG2 cells, and these were 2-propylheptanoic acid (2-PHP), 2-Ethylheptanoic acid, 2-propylhexanoic acid, 2-ethylhexanoic acid and valproic acid (VPA), with VPA and 2-PHP being the two most potent analogues in this group.

In this paper, we have used transcriptional profiling in *vitro* systems as a read out to determine the biological activity associated with a particular set of related chemicals and with that, substantiate the suitability of a chemical rich in safety data and support a RAX assessment of a target chemical. Two case studies are presented: the first one with 4 linear chain n-alkyl parabens where propylparaben (PP) is the target chemical, while methyl-(MP), ethyl-(EP), and butylparaben (BP) are the suitable structural analogs, as well as the main metabolite of these parabens, p-hydroxybenzoic acid (pHBA). The second case is with caffeine (CA) being the target chemical and three of its main metabolites theophylline (TP), theobromine (TB) and paraxanthine (PX) as the suitable structural analogs. The indicated structural analogs of each target chemical were identified by expert judgment following the process for evaluating analogs for use in Structure-Activity Relationship (SAR) described by Wu et al. (2010). Even though each target chemical has a robust safety data set derived from animal studies performed prior to the testing ban, the assumption was that there were data gaps for the target chemical of both case studies. The data gap selected for PP was the reproductive and developmental toxicity, and for CA was its systemic toxicity. Thus, the goal of using transcriptional profiling of cultured cells was to identify potential concerns for these endpoints, for each target chemical, and at the same time, identify a biologically similar chemical for RXA and fill the appropriate data gap.

In this study, the transcriptional profile of each analog for each case study was evaluated in four cell types: MCF7, HepG2, A549 and iCell Cardiomyocytes, and compared to the target of the respective case study. This comparison was used to determine whether the biological activity for sets of structurally-related chemicals is comparable. Our results clearly show that the transcriptional profile elicited by each of the parabens shares a high degree of similarities across the group members, with the highest similarity observed between BP and PP. Similar results were obtained in the case of CA and its metabolites, where the transcriptional profile of each methylxanthine has significant concordance with each other, with the highest transcriptional profile similarity between CA and TP. Pathway enrichment analysis (MSigDB v7.4) of the transcriptional profile for each chemical in both cases studies indicated a significant overlap in the regulated pathways identified by analyzing the transcriptional profiles of up- and down-regulated genes across the two chemical groups, with the highest similarities between PP and BP, and CA and TP respectively, supporting the validity of the read across among the group, with BT and TP being the closest structural and biological analogue for PP and CA, respectively. In all, our results support the use of transcriptional profiling in chemical-sensitive cultured cells to define the biological activity of chemical classes and to better define chemical analogs, and thus increase the confidence in a RAX approach by strengthening the traditional structure-based approaches useful in hazard assessment.

## Methods

### Chemicals and reagents

Propylparaben (CAS# 94-13-3), methylparaben (CAS# 99-76-3), ethylparaben (CAS# 120-47-8), butylparaben (CAS# 94-26-8) p-hydroxybenzoic acid (CAS# 99-96-7), caffeine (CAS# 58-08-2), theophylline (CAS# 58-55-9), theobromine (CAS# 83-67-0), paraxanthine (CAS# 611-59-6), trichostatin A (CAS# 58880-19-6), 3-(4,5-dimethylthiazol-2-yl)-2,5-diphenyltetrazolium bromide (CAS# 298-93-1) and dimethyl sulfoxide (67-68-5) were all obtained from Sigma-Aldrich (St Louis, MO).

### Concentration and time point selection

For each chemical tested in this case study, a cytotoxicity assessment was performed in one of the cell lines used in the experiments (i.e. MCF7, HepG2 or A549 cells) using increased concentrations of the chemicals up to a concentration that resulted in some toxicity, but no more than 10% cytotoxicity as measured by the 3-(4,5-dimethylthiazol-2-yl)-2,5-diphenyltetrazolium bromide (MTT) assay according to Mosmann (1983). In our experience, the cytotoxic effects of any given chemical can be determined in any of the indicated cell types, and these cells do not display a particular sensitivity to cytotoxic effects when compared among each other. The highest concentration of each chemical that elicited no more than 10% cytotoxicity, determined in this preliminary experiment, was selected as the highest concentration (D1) to be evaluated in each of the 4 cell lines: MCF7, HepG2, A549 cells (obtained from American Type Culture Collection, ATCC), and iCell Cardiomyocytes (obtained from Fujifilm/Cellular Dynamics). To determine the transcriptional profile associated with the exposure to each chemical, each cell line was treated with D1 and two dilutions of D1 (D2 and D3), for a total of 3 concentrations for each chemical for each cell line (Tables 1 and 3) and compared to the appropriate control (cell line treated with the appropriate concentration of vehicle, dimethyl sulfoxide, DMSO). The concentration of DMSO of 0.1% was maintained constant across the different chemicals and cells evaluated, including the vehicle controls. In the parabens case study, the response to each chemical was evaluated at 1, 50 and 500 μM; while in the methylxanthines case study the response to each chemical was evaluated at 50, 500 and 1,000 μM. For every chemical and cell type evaluated, 4 biological replicas were generated.

**TABLE 1:** Number of genes whose expression was significantly (FDR<0.05, FC ± 1.2) modified by MP (CAS# 99–76-3), EP (CAS# 120–47-8), BP (CAS# 94–26-8) and PP (CAS# 94-13-3), as well as the main metabolite of these parabens, pHBA (CAS# 99-96-7), in each of the cell types evaluated.

Chemical	Concentration (μM)	Number of genes whose expression was modified by each chemical^a^			
		A549	iCAR	HepG2	MCF7
Methylparaben	500	25	4	17	637
Methylparaben	50	974	1	1	1
Methylparaben	1	0	0	1	0
Ethylparaben	500	31	5,436	22	577
Ethylparaben	50	0	0	1	47
Ethylparaben	1	0	1	1	0
Butylparaben	500	5,817	3,767	3,988	5,111
Butylparaben	50	5	2	3	135
Butylparaben	1	0	1	0	0
Propylparaben	500	182	56	6	1755
Propylparaben	50	1	1	1	161
Propylparaben	1	0	0	0	0
p-Hydroxybenzoic acid	500	0	8	0	17
p-Hydroxybenzoic acid	50	0	2	3	1
p-Hydroxybenzoic acid	1	0	0	0	1

^a^ **FDR <0.05**.

The exposure time for each chemical and all the 4 cell lines was 6 h. This time has been selected in order to both obtain a signature related to the direct mechanism of action (molecular initiating event, MIE) of the chemical being evaluated and to maintain consistency with earlier experiments (Lamb et al., 2006; De Abrew, et al., 2016; De Abrew, et al., 2019). Other studies have shown the importance of using early response genes in predictive toxicology (Zhang et al., 2014). This time point was also found to be more informative than later time points by Lamb et al. (2006). Thus, whole cell lysates were obtained after 6 h of exposure to each chemical or the appropriate vehicle (controls) for transcriptional profiling, using the lysis buffer recommended by BioSpyder (provider of the TempO-seq platform). In every experiment, each cell type was also exposed to 0.1 μM Trichostatin A, used as positive control.

### Gene expression profiling

The transcriptional response to the exposure to each chemical was evaluated in 4 cell lines: MCF-7, A549, HepG2 and iCell Cardiomyocytes cultured using recommended culture protocols and reagents (ATCC or Fujifilm/Cellular Dynamics, respectively). Each cell type displays a specific phenotype from the representative target organ, including an endocrine-responsive (MCF-7), liver-derived (HepG2), lung-derived (A549) and an electrically active cell type (iCell Cardiomyocytes) and are terminally differentiated. The use of these 4 cell types allows for a broader “biological coverage” to determine the potential effect on gene expression of any given chemical being evaluated. Cells were seeded on 96 well plates and treated with 3 concentrations of each chemical or vehicle (DMSO) for 6 h. Treatments were randomized across the plate to minimize batch effect and dispensed using an Andrew System 1000G liquid handling robot (Andrew Alliance, Waltham, MA). Following 6 h treatment, cell lysates were harvested according to a protocol provided by Biospyder (TempO-Seq Workflow—BioSpyder) and then stored at −80°C until analysis. Each experiment was performed in 4 biological replicas, with the cells for each replicate treated and harvested on separate days, to better represent 4 biological replicas. Cell lysate plates were shipped frozen to BioSpyder (BioSpyder Technologies, Inc., Carlsbad, CA) and Tempo-Seq human whole transcriptome (version 2.0) assay was performed at BioSpyder as previously described (Yeakley et al., 2017).

The complete gene expression data have been deposited in the National Center for Biotechnology Information Gene Expression Omnibus (GEO) (Edgar et al., 2002) and are accessible through GEO Series accession number GSE218902.

### Gene expression statistical analysis

The transcriptional profiling data, consisting of Log2 transformed gene expression data, was obtained from the appropriate raw data provided by BioSpyder. FASTQ files from the Illumina standard sequencing instrument software were provided by the vendor. Each FASTQ file was aligned using the Bowtie algorithm by vendor to generate a count table with each column representing a sample and each row representing a gene. The count table generated from each cell line was used for gene expression analysis using DESeq2 v1.30.0 (Love et al., 2014) package in R software (v4.0.3). Probes with count of 5 or more in at least 3 samples were kept in prefiltering step. DESeq2 default parameters and size factor estimation were used for normalization. Negative binomial model was used for computing the differential expressed genes (DEGs) compared to vehicle controls. Fold change shrinkage was applied for each concentration *versus* related controls to compute moderated L2FC values for each probe. Probes with adjusted *p*-value (FDR) ≤ 0.05 were considered as differentially expressed. The 100 most up- and down-regulated genes with smallest *p* values induced by each chemical treatment (concentration vs. control within each cell line) were selected as gene signatures to query the connectivity map (cMAP) database.

### Gene ontology and canonical pathway analysis

Gene Ontology (GO) and canonical pathways of DEGs were analyzed in the Molecular Signature Data Base (MSigDB version 7.1) of the Gene Set Enrichment Analysis (GSEA) website (http://www.gsea-msigdb.org) (Subramanian et al., 2005). The separated list of up-regulated and down-regulated DEGs identified following chemical exposure were used to assess which pathways were affected by each chemical using MsigDB. In this case, each transcriptional profile was evaluated using the 50 gene sets under the Hallmark gene sets (H) using the appropriate human gene symbols for each gene being queried. Enrichment of GO terms, KEGG pathways, and Reactome pathways was considered significant when the FDR q value was less than 0.05 and at least 5 up- or down-regulated genes were part of the pathway.

### Connectivity map (cMAP) analysis

In order to identify similarities in biological activity across the different chemicals evaluated in the different cell lines, we used the cMAP approach, originally described by Lamb et al. (2006), and the updated Library of Network-Based Cellular Signatures (LINCS; Subramanian et al., 2017) database using the CLUE Touchstone 1.1.1.43 ((clue.io)) database. This approach allows to validate the transcriptional profiling data as well as to discover connections between the chemicals being evaluated and chemicals already evaluated using pattern-matching recognition of their expression profiles. This database includes 8,969 well studied and annotated small-molecule compounds and genetic reagents tested in nine human cell lines. After raw data preprocessing, the log2 gene expression data from each chemical, at each concentration, was used to calculate fold-change for each gene evaluated and whose expression is affected by each chemical treatment with respect to the average control of the corresponding batch. The fold-change of each gene was used to produce a gene expression profile or signature for the chemical being evaluated (at each concentration tested) using the standard method described in the original cMAP paper (Lamb, et al., 2006). To generate signatures for each chemical (independently for each concentration), a two-sample *t*-test paired for instances tested in the same batch is run using the Limma software (Smyth, 2005; Wettenhall et al., 2008). A 5% false-discovery rate (FDR) cut-off is used to generate up-regulated (positive fold-change) and down-regulated (negative fold-change) signatures. This gene expression signature of each chemical that generated significant gene expression changes (at any concentration), combining both up-regulated and down-regulated gene expression changes, was compared with each signature represented in the cMAP database at the Broad Institute ((clue.io)) and scored to determine the degree of similarity between the signature being evaluated and the “matched” signature of chemicals identified in the data base. The connectivity score provides three measures of confidence: 1) a nominal *p*-value derived by comparing the similarity between the query and reference signature, using the Kolmogorov-Smirnov enrichment statistic (Subramanian et al., 2005), to a null distribution of random queries; 2) a false discovery rate (FDR) that adjusts the *p*-value to account for multiple hypothesis testing; and 3) Tau (τ), which compares an observed enrichment score to all others in the database. cMAP scores range from +100, representing similar gene expression signature between the two chemicals being compared, to −100 representing opposite signatures. The premise in this analysis is that transcriptional signatures with high similarity (cMAP scores close to +100) represent similar biological activity. We also compared the transcriptional response of the active chemicals from this case study to each other, as well as to other chemicals we have evaluated in our lab (De Abrew et al., 2019).

## Results

### Parabens

**FIGURE 1:**
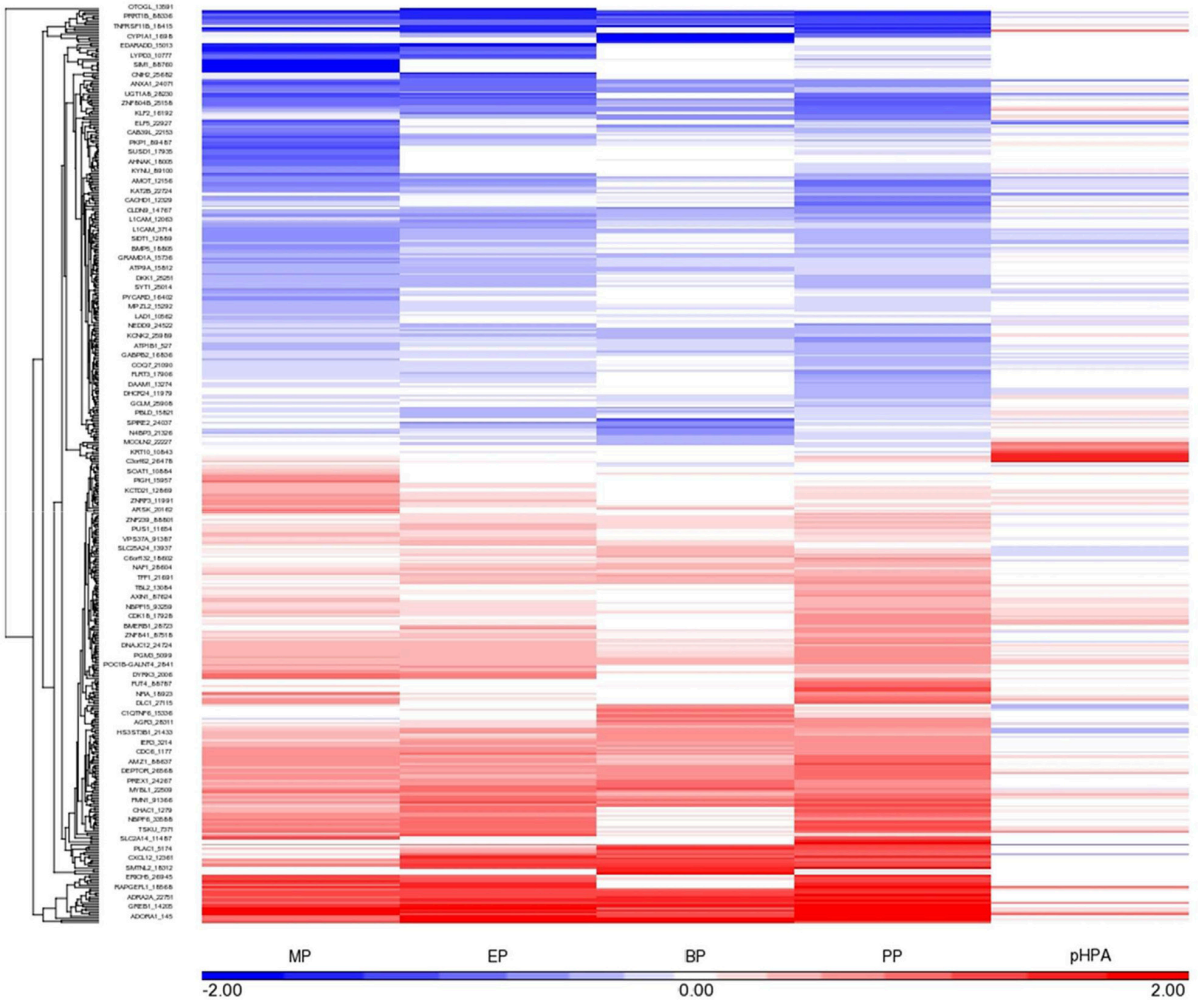
Gene expression profile elicited by MP, EP, BP and PP, as well as the main metabolite of these parabens, pHBA (at the highest concentrations tested, 500 μM, except for BP, shown 50 μM response) in MCF-7 cells. The genes showing a robust response after exposure to each paraben (FDR<0.05 and at least 1.2 fold change, up- or down-regulated) are shown in this diagram. Each cell is represented as a color-coded rectangle in which the color indicates the expression value (fold change) of unaffected (white), up-regulated (red) or down-regulated (blue) genes. The hierarchical clustering is based upon the concentration-response, and the positioning has been established according to the similarity in response of these genes to each paraben evaluated. The length of the lines in the tree indicates the similarity in regulatory pattern for each gene, with shorter length indicating more similarity.

The number of genes whose transcriptional response was affected by exposure to MP, EP, BP and PP, as well as by its main metabolite pHBA, at each of the concentrations tested, on each of the cell lines assessed, and with an FDR≤0.05, is shown in Table 1. Each of the parabens elicited changes in the expression of a number of genes, as compared to controls, particularly at the highest concentration tested. The most robust and consistent response was observed in the MCF7 cells (Figure 1). Since the transcriptional response of the MCF7 cells to 500 μM BP was too strong (judged by the number of genes affected), as compared to the other 3 parabens (Table 1) and to better visualize the similarities across the group, in Figure 1 the response of the cells to 50 μM BP was included and compared to the response of the cells to the highest concentrations of the other parabens. The transcriptional profile elicited by each of the parabens shares a high degree of similarities across the group of chemicals. Comparison of the MCF7 cells’ response to the group, resulted in the identification of 133 common genes whose expression is modified by each of the parabens in a significant manner in the same direction. pHBA shared only 17 genes in common with the group. Comparing the transcriptional response to each paraben, at the highest concentration tested (500 μM), with the response to PP we determined that the highest number of genes commonly affected by the parabens was found between BP and PP, where 634 genes were affected in the same direction by PP and BP, of which the expression of 319 genes was up-regulated while the expression of 315 genes was down-regulated. Doing the same comparisons using the percent of genes that are commonly affected by the exposure to the PP and the other parabens, the highest similarity in the response was also between PP and BP, with 36% common genes affected by both parabens. The overlap of the response between MP and PP is 20%, while between ET and PP is 24%.

Pathway enrichment analysis (MSigDB v7.5.1) of the transcriptional profile for each paraben indicated a significant overlap in the regulated pathways across the four parabens. For this analysis the up-regulated and down-regulated gene sets were analyzed separately. The top individual pathways affected by exposure to BP and PP in MCF7 cells is shown in Table 2. The top Hallmark pathways being regulated by genes whose expression was up-regulated by these two parabens are: estrogen response early and late, and TNFA signaling via NFKB. While the top Hallmark pathways that are regulated by genes whose expression was down-regulated by both BP and PP are: G2M checkpoint, bile acid metabolism, and hedgehog signaling. These pathways were also enriched with the individual sets of genes affected by BP (at 50 or 500 μM) or PP were used for the analysis.

**TABLE 2:** Pathway enrichment analysis using the transcriptional profile identified in the MCF-7 cells exposed to BP and PP at 500 μM. For this analysis the Molecular Signatures Database (MSigDB v7.5.1) was utilized, and only the top enriched gene sets identified with the up-regulated or down-regulated genes (FDR <0.05, and fold change of 1.2 >) by both BP and PP (common genes affected in the same directions) are shown in the table.

Regulated pathways by up-regulated genes by BP and PP at 500 μM in MCF7 cells				
Gene Set Name (# Genes (K))	Description	# Genes in Overlap (k)	*p*-value	FDRq-value
HALLMARK_ESTROGEN_RESPONSE_EARLY (200)	Genes defining early response to estrogen	25	7.63 e-37	3.81 e-35
HALLMARK_ESTROGEN_RESPONSE_LATE (200)	Genes defining late response to estrogen	23	5.06 e-33	1.26 e-31
HALLMARK_TNFA_SIGNALING_VIA_NFKB (200)	Genes regulated by NF-kB in response to TNF (GeneID = 7,124)	7	3.92 e-7	6.54 e-6
HALLMARK_IL2_STAT5_SIGNALING (199)	Genes up-regulated by STAT5 in response to IL2 stimulation	5	9.4 e-5	1.18 e-3
HALLMARK_UNFOLDED_PROTEIN_RESPONSE (113)	Genes up-regulated during unfolded protein response, a cellular stress response related to the endoplasmic reticulum	4	1.32 e-4	1.32 e-3
HALLMARK_UV_RESPONSE_UP (158)	Genes up-regulated in response to ultraviolet (UV) radiation	4	4.74 e-4	3.64 e-3
HALLMARK_APOPTOSIS (161)	Genes mediating programmed cell death (apoptosis) by activation of caspases	4	5.09 e-4	3.64 e-3
HALLMARK_EPITHELIAL_MESENCHYMAL_TRANSITION (200)	Genes defining epithelial-mesenchymal transition, as in wound healing, fibrosis and metastasis	4	1.14 e-3	5.19 e-3
HALLMARK_G2M_CHECKPOINT (200)	Genes involved in the G2/M checkpoint, as in progression through the cell division cycle	4	1.14 e-3	5.19 e-3
HALLMARK_GLYCOLYSIS 200)	Genes encoding proteins involved in glycolysis and gluconeogenesis	4	1.14 e-3	5.19 e-3
**Regulated pathways by down-regulated genes by BP and PP at 500 μM in MCF7 cells**				
**Gene Set Name** [**# Genes (K)]**	**Description**	**# Genes in Overlap (k)**	*p* **-value**	**FDRq-value**
HALLMARK_G2M_CHECKPOINT (200)	Genes involved in the G2/M checkpoint, as in progression through the cell division cycle	4	9.79 e-5	4.9 e-3
HALLMARK_BILE_ACID_METABOLISM (112)	Genes involve in metabolism of bile acids and salts	3	3.33 e-4	8.33 e-3
HALLMARK_HEDGEHOG_SIGNALING (36)	Genes up-regulated by activation of hedgehog signaling	2	8.58 e-4	1.43 e-2
HALLMARK_HEME_METABOLISM (200)	Genes involved in metabolism of heme (a cofactor consisting of iron and porphyrin) and erythroblast differentiation	3	1.78 e-3	2.23 e-2

**FIGURE 2:**
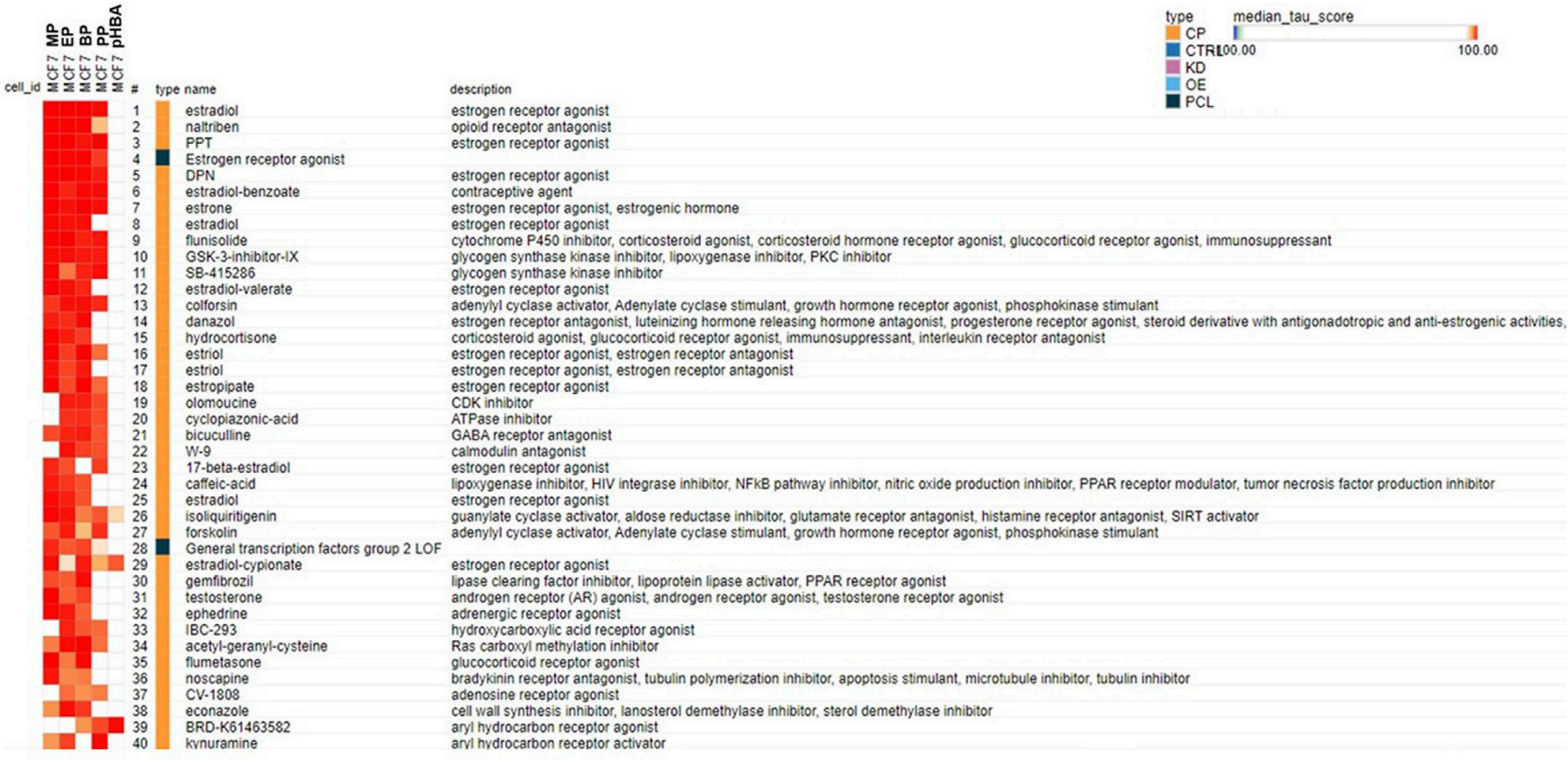
Connectivity map analysis using the most robust responsive genes identified in the MCF-7 cells exposed to MP, EP, BP and PP (at the highest concentrations tested, 500 μM) in the Clue Touchstone database 1.0 set. The genes showing the most robust response after exposure to each chemical (top 100 up- or down-regulated genes with the smallest *p* values) were used for the cMAP analysis. Only the top 40 chemicals identified from the Broad Institute’s CLUE Touchstone data base, with similar transcript profile to each chemical (positive connection) are shown. The same chemical could be listed multiple times (i. e. estradiol), since the individual transcript profile was obtained in different cell types, and or concentrations. The solid blue block on the “type” column represents connectivity to the indicated class of chemicals (i.e. Estrogen receptor agonists).

The similarity in biological activity among the four parabens is also shown in the cMAP analysis using the most robust responsive genes identified in the MCF-7 cells exposed to MP, EP, BP or PP (at the highest concentrations tested, 500 μM) in the Clue Touchstone database 1.0 set (Figure 2). This similarity is not existent between any paraben and their metabolite pHBA. The transcriptional profile elicited by each paraben is highly similar to the one elicited by chemicals known to act as estrogen receptor agonists (i.e. estradiol, estradiol benzoate and estrone).

### Methylxanthines

**FIGURE 3:**
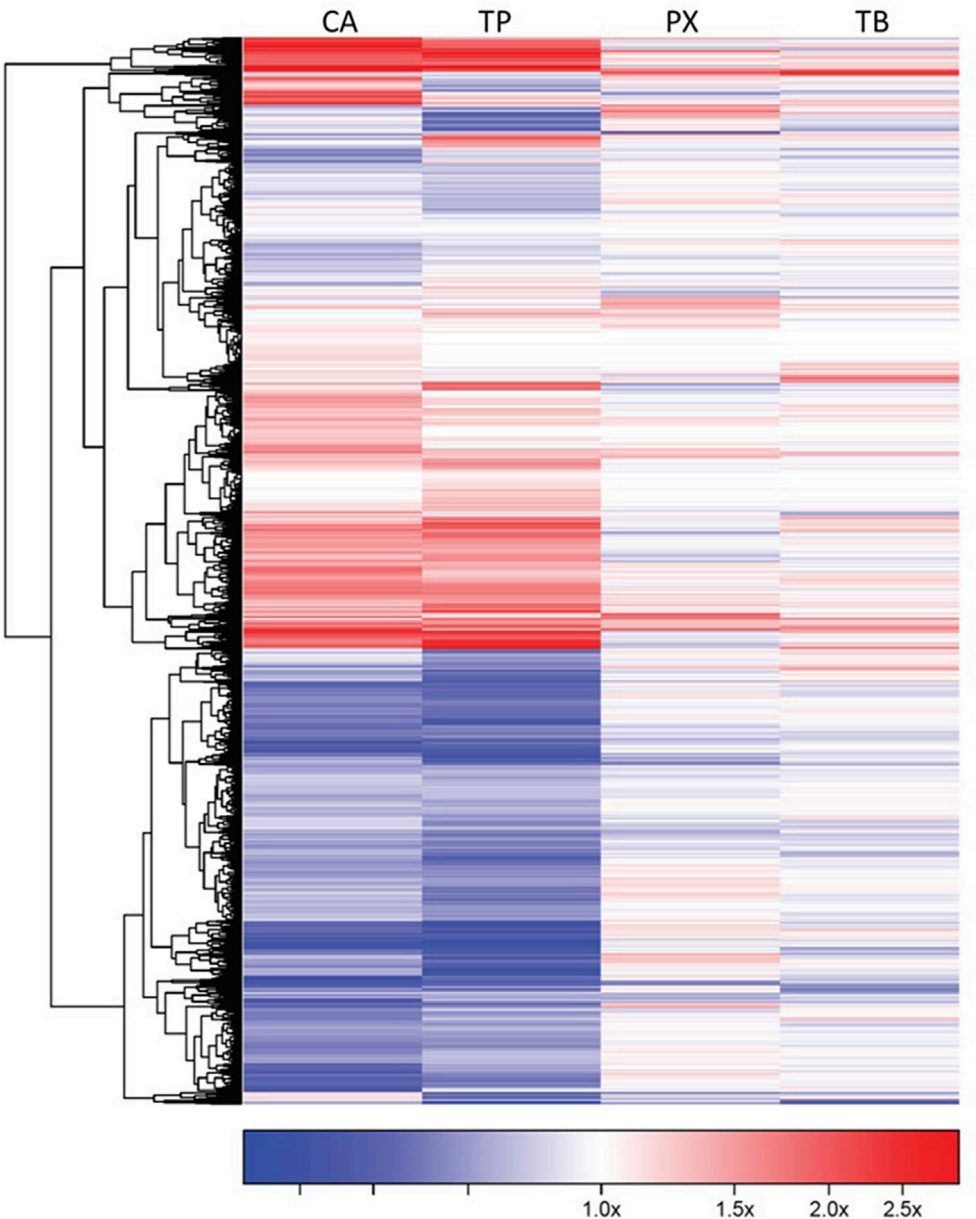
Gene expression profile of iCell-Cardiomyocytes exposed to CA, TP, PX or TB (1,000 μM). The genes showing a robust response after exposure to each methylxanthine (FDR<0.05 and at least 1.2 fold change, up- or down-regulated) are shown in this diagram. Each cell is represented as a color-coded rectangle in which the color indicates the expression value (fold change) of unaffected (white), up-regulated (red) or down-regulated (blue) genes. The hierarchical clustering and the positioning has been established according to the similarity in response of these genes to each chemical evaluated. The length of the lines in the tree indicates the similarity in regulatory pattern for each gene, with shorter length indicating more similarity.

The number of significant genes (FDR ≤0.05) whose transcriptional response was affected by exposure to CA, TB, TP or PX, at each of the concentrations tested, on each of the cell lines assessed, is shown in Table 3. All the cell types evaluated respond to each methylxanthine, showing significant changes in the expression of multiple genes, as compared to controls, particularly at the highest concentration tested (Figure 3). CA and TP seem to be the most active, with robust and consistent response in the four cell types. Comparing the transcriptional response across the four cell types, the response of the cardiomyocytes is the most similar between CA and TP, with 604 genes affected in the same direction by these two methylxanthines. Interestingly, the majority of the genes whose expression is affected by both CA and TP are up-regulated (545 genes), the same results were also determined in the transcriptional profile elicited by CA or TP in the four cell types exposed to these methylxanthines, the up-regulated genes outnumber the down-regulated genes. Comparing the response of the cardiomyocytes to the four methylxanthines based on the percent of genes that are commonly affected by the exposure, the highest similarity in the response was also between TP and CA, with 61.8% common genes whose transcriptional response was affected in the same direction by both methylxanthines.

**TABLE 3:** Number of genes whose expression was significantly (FDR<0.05, FC ± 1.2) (CAS# 58–08-2), TB (83–67-0), TP (CAS# 58–55-9) or PX (CAS# 611–59-6) in each of the cell types evaluated.

		Number of genes whose expression was modified by each chemical^a^			
Chemical	Concentration (μM)	A549	iCAR	HepG2	MCF7
Caffeine	1,000	60	977	266	480
Caffeine	500	35	0	23	274
Caffeine	50	0	3	0	1
Theobromine	1,000	77	23	157	31
Theobromine	500	2	10	26	1
Theobromine	50	0	2	0	3
Theophylline	1,000	225	1,060	609	350
Theophylline	500	42	5	118	40
Theophylline	50	3	0	271	0
Paraxanthine	1,000	45	28	283	159
Paraxanthine	500	3	3	28	15
Paraxanthine	50	36	0	0	1

^a^ FDR <0.05.

The up-regulated and down-regulated gene sets identified for each of the methylxanthines were used separately for pathway enrichment analysis (MSigDB v7.5.1) to identify the pathways that were particularly affected by each set of genes. This analysis was done with the gene sets for each methylxanthine independently, as well as with the set of genes commonly affected by both CA and TP. When the common set of genes whose expression was affected by both CA and TP was used for the pathway enrichment analysis, the highest similarity in biological activity was found between the individual responses to CA or TP (Table 4). The top Hallmark pathways that are most regulated by genes up-regulated by these two methylxanthines in cardiomyocytes are: TNFA signaling *via* NFKB, hypoxia, UV response and mitotic spindle, while the most regulated pathways by genes down-regulated are: E2F targets, DNA repair, apoptosis and interferon gamma response.

**TABLE 4:** Pathway enrichment analysis using the transcriptional profile identified in the cardiomyocytes exposed to CA or TP at 1,000 μM individually A) or by both CA and TP B). For this analysis the Molecular Signatures Database (MSigDB v7.5.1) was utilized, and only the top enriched gene sets identified with the up-regulated or down-regulated genes (FDR <0.05, and fold change of 1.2 >) either for CA or TP, individually or by both CA and TP (common genes affected in the same directions) are shown in the tables.

Regulated pathways by up-regulated genes by both CA and TP at 1,000 μM in cardiomyocytes								
Gene Set Name (# Genes (K))		Description		# Genes in Overlap (k)		*p*-value		FDRq-value
HALLMARK_TNFA_SIGNALING_VIA_NFKB (200)		Genes regulated by NF-kB in response to TNF (GeneID = 7,124)		15		2.96 e-8		1.48 e-6
HALLMARK_HYPOXIA (200)		Genes up-regulated in response to low oxygen levels (hypoxia)		12		7.3 e-6		1.83 e-4
HALLMARK_UV_RESPONSE_DN (144)		Genes down-regulated in response to ultraviolet (UV) radiation		9		7.36 e-5		1.23 e-3
HALLMARK_MITOTIC_SPINDLE (199)		Genes important for mitotic spindle assembly		10		1.82 e-4		1.9 e-3
HALLMARK_ESTROGEN_RESPONSE_EARLY (200)		Genes defining early response to estrogen		10		1.9 e-4		1.9 e-3
HALLMARK_ANDROGEN_RESPONSE (101)		Genes defining response to androgens		7		2.46 e-4		2.05 e-3
HALLMARK_MTORC1_SIGNALING (200)		Genes up-regulated through activation of mTORC1 complex		9		8.38 e-4		5.24 e-3
HALLMARK_P53_PATHWAY (200)		Genes involved in p53 pathways and networks		9		8.38 e-4		5.24 e-3
HALLMARK_HEDGEHOG_SIGNALING (36)		Genes up-regulated by activation of hedgehog signaling		4		9.44 e-4		5.24 e-3
HALLMARK_PROTEIN_SECRETION (96)		Genes involved in protein secretion pathway		6		1.17 e-3		5.85 e-3
HALLMARK_IL2_STAT5_SIGNALING (199)		Genes up-regulated by STAT5 in response to IL2 stimulation		8		3.23 e-3		1.39 e-2
HALLMARK_KRAS_SIGNALING_UP (200)		Genes up-regulated by KRAS activation		8		3.33 e-3		1.39 e-2
HALLMARK_PI3K_AKT_MTOR_SIGNALING (105)		Genes up-regulated by activation of the PI3K/AKT/mTOR pathway		5		9.41 e-3		3.41 e-2
HALLMARK_ANGIOGENESIS (36)		Genes up-regulated during formation of blood vessels (angiogenesis)		3		9.54 e-3		3.41 e-2
HALLMARK_EPITHELIAL_MESENCHYMAL_TRANSITION (200)		Genes defining epithelial-mesenchymal transition, as in wound healing, fibrosis and metastasis		7		1.18 e-2		3.93 e-2
HALLMARK_APOPTOSIS (161)		Genes mediating programmed cell death (apoptosis) by activation of caspases		6		1.44 e-2		4.51 e-2
**Regulated pathways by down-regulated genes by both CA and TP at 1,000 μM in cardiomyocytes**								
**Gene Set Name (# Genes K))**	**Description**		**# Genes in Overlap k)**		*p* **-value **		**FDRq**-**value **	
HALLMARK_E2F_TARGETS (200)	Genes encoding cell cycle related targets of E2F transcription factors		4		2.02 e-4		1.01 e-2	
HALLMARK_DNA_REPAIR (150)	Genes involved in DNA repair		3		1.34 e-3		2.74 e-2	
HALLMARK_APOPTOSIS (161)	Genes mediating programmed cell death (apoptosis) by activation of caspases		3		1.64 e-3		2.74 e-2	
HALLMARK_INTERFERON_GAMMA_RESPONSE (200)	Genes up-regulated in response to IFNG (GeneID = 3,458)		3		3.04 e-3		3.04 e-2	
HALLMARK_P53_PATHWAY (200)	Genes involved in p53 pathways and networks		3		3.04 e-3		3.04 e-2	

**FIGURE 4:**
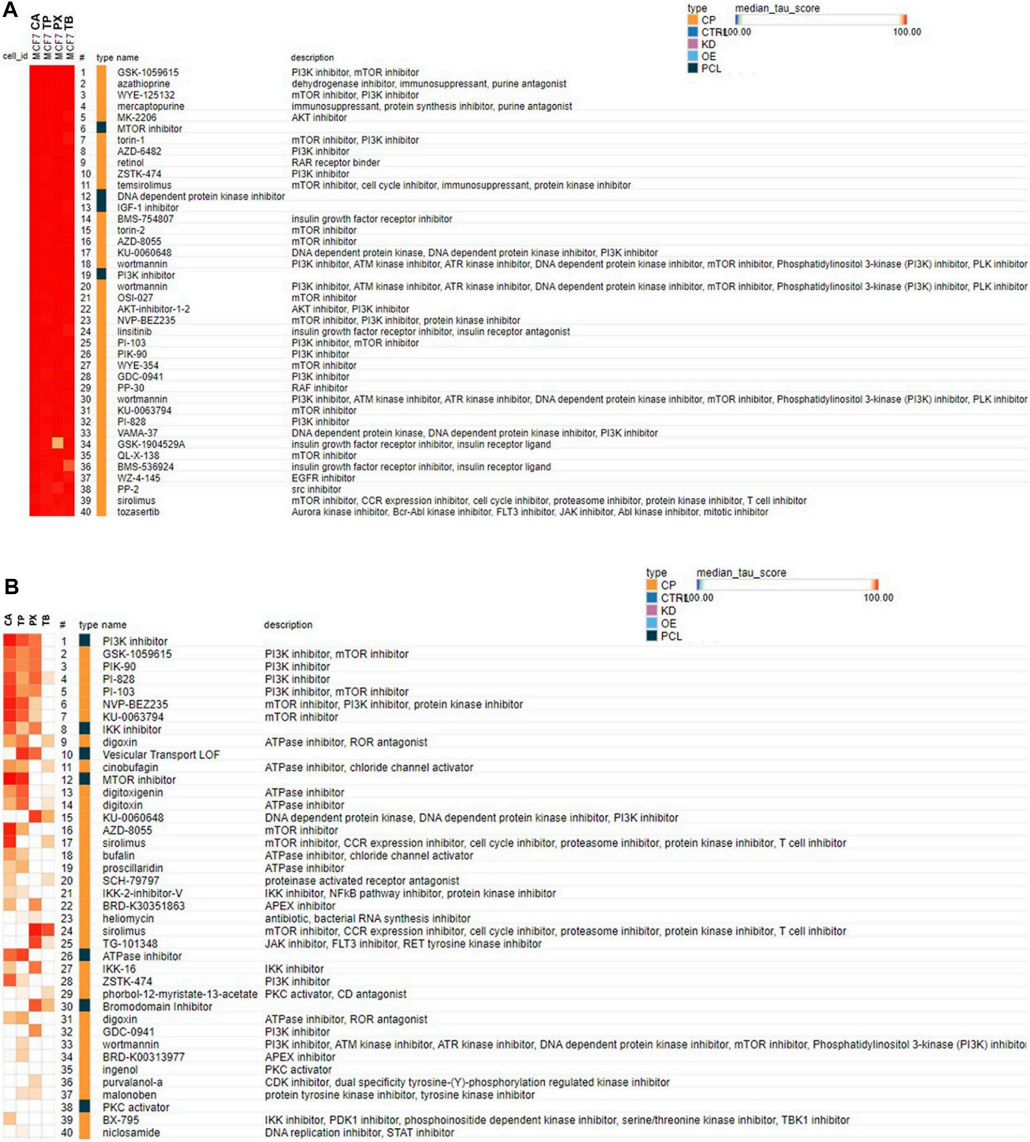
Connectivity map analysis using the most robust responsive genes identified in the MCF-7 cells **(A)** or cardiomyocytes **(B)** exposed to CA, TP, PX or TB (at the highest concentrations tested, 1,000 μM) in the Clue Touchstone database 1.0 set. The genes showing the most robust response after exposure to each chemical (top 100 up- or down-regulated genes with the smallest *p* values) were used for the cMAP analysis. Only the top 40 chemicals identified from the Broad Institute’s CLUE Touchstone data base, with similar transcript profile to each chemical (positive connection) are shown. The same chemical could be listed multiple times (i. e. wortmannin), since the individual transcript profile was obtained in different cell types, and or concentrations.

For the cMAP analysis, the transcriptional profile elicited by each of the methylxanthines in MCF-7 cells as well as in cardiomyocytes was used. The cMAP analysis of the MCF-7 cells set of data for the 4 methylxanthines (Figure 4A) indicates that this data seems to have better connectivity than the data from the cardiomyocytes (Figure 4B), this could be due to the fact that cardiomyocytes are not represented in the Clue database while the MCF-7 cells are. The similarity in biological activity among the four methylxanthines is shown in the connectivity map analysis using the most robust responsive genes identified in either the MCF-7 cells or the cardiomyocytes exposed to TP, PX or TB (at the highest concentrations tested, 1,000 μM) in the Clue Touchstone database 1.0 set (Figure 4). Based on the connectivity results, it is clear that the transcriptional profile elicited by each methylxanthine is similar among each other, however the highest similarity is between CA and TP. The transcriptional response to both CA and TP in either MCF-7 cells (Figure 4A) or cardiomyocytes (Figure 4B) is very similar to the one elicited by chemicals known to act as inhibitors of the PI3K and mTOR pathway, as well as with ATPase and inhibitors of nuclear factor kappa-B kinase (IKK) inhibitors.

## Discussion

Traditionally a RAX assessment is based on the premise that chemicals with similar structure will either have similar reactivity, and thus have similar biological activity or be metabolized to the same active intermediate. Much work has been done to identify the chemical features that convey analog suitability, including structure, metabolism, reactivity and physicochemical properties (e.g., Wu et al., 2010; Lester and Yan, 2021). However, even if all these features are considered to select the best candidate(s) to RAX, the similarity in biological activity of this chemical has to be substantiated. There are multiple instances where empirical data shows that similar compounds do not interact with the target macromolecule(s) in similar ways and thus display differences in biological activity (Martin et al., 2002; Redžepović and Furtula, 2022). Different approaches have been suggested for demonstrating similar biological activity, with transcriptomic profiling showing great potential (De Abrew et al., 2016; De Abrew et al., 2019; Harrill et al., 2021). The comprehensive transcriptional profile associated with the exposure of a sensitive cell type to chemicals being considered for RAX can be used to define their biological activity, determine the mode of action used to cause a potential adverse event, and thus help to distinguish the most similar analogues to support the RAX. In this study we have substantiated the value of using transcriptional profiling to identify biologically similar chemicals within a group to be usable in a RAX assessment with two unrelated set of chemicals: four short alkyl chain parabens and caffeine and three of its main metabolites.

### Short alkyl chain parabens case study

For the purposes of the case study, we assumed there was a data gap for PP with respect to reproductive and developmental toxicity. A full description of this case study has been recently published (Ouedraogo et al., 2022). A category approach was taken for its assessment, defining PP as the target chemical and MP, EP and BP identified as the most relevant structural analogues from which to read across. In our assessment, we included pHBA since it is the major and primary metabolite of the four parabens after ester hydrolysis and could be involved in the elicitation of the biological activity of these short alkyl chain parabens. The objective was to use transcriptional profiling to demonstrate the biological activity similarities between the category members and determine which of the parabens was the most suitable analogue to read across in the assessment of the target PP. Each of the parabens elicited changes in the expression of a number of genes, as compared to controls, particularly at the highest concentration tested, and this transcriptional profile is unique for each of the parabens; however there is a common set of genes whose expression is modified by the four parabens assessed here. By looking into the number of genes whose expression is significantly affected by each of the parabens, as a potency read-out, it can be concluded that the parabens exhibit a predictable potency trend in observed effects across category members with increasing alkyl chain length: MP < EP < PP < BP. Importantly, the transcriptional profile elicited by each of the parabens tested here shares a high degree of similarities across the category members. We identified 133 genes whose expression is modified by each of the parabens in a significant manner in the same direction. pHBA elicited significant gene expression changes at the highest concentration evaluated, however, these changes are for the most different than the ones elicited by any of the parabens. The pHBA transcriptional profile only shares 17 genes in common with the transcriptional response to the four parabens evaluated here. The highest number of genes commonly affected by the parabens was found between BP and PP, where 634 genes were affected in the same direction. The analysis of the transcriptional response of the MCF7 cells to MP, EP or BP to the one elicited by PP, at the same concentrations, on the bases of percent of overlap also supports our conclusion that the highest similarity in the transcriptional response is between BP and PP.

To determine the most relevant biological activities of each of the parabens in the case study, the transcriptional profiles were analysed for pathway enrichment. Pathway enrichment analysis of the transcriptional profile for each paraben indicated a significant overlap in the regulated pathways by either the up-regulated or the down-regulated genes across the four parabens. This analysis provides evidence of strong concordance in the biological activity of the short alkyl chain parabens and similar potential mode of action. The highest degree of similarity at the pathway level was found between BP and PP, the top Hallmark pathways that are most regulated by up-regulated genes by these two parabens are: estrogen response early and late, TNFalpha signalling *via* NFKB and IL2-STAT5-signaling. While the top Hallmark pathways that are most regulated by down-regulated genes are: apical junction, NOTCH signalling, myogenesis, and hedgehog signalling. These pathways’ similarities further support the conclusion that these two parabens are the most similar structural and biological analogues among the group and thus the data from BP can be used to read-across and fill the data gap for *p*P. The cMAP analysis of the transcriptional response for PP and BP also points toward an estrogenic response after exposure to these parabens, particularly pointing towards an estrogen receptor agonist activity. The pathway enrichment analysis, together with the cMAP results indicate that the parabens, particularly at high concentrations, might have the ability to impact the estrogen pathway by modifying the expression of genes associated with this pathway, and thus potentially could modify the estrogenic response. The results are consistent with previous reports indicating that MP, EP, PP and BP have a weak estrogenic activity *in vitro* (Routledge et al., 1998) as well as *in vivo* (Routledge et al., 1998; Hossani et al., 2000; Lemini et al., 2003; Lemini et al., 2004). The results from an *in vivo* assay established as a reliable method to determine estrogenic activity for chemicals of concern, the rat uterotrophic assay, indicate that at the most MP, EP and BP have a weak estrogenic activity *in vivo* (Routledge et al., 1998; Hossaini et al., 2000; Lemini et al., 2004); while PP does not display estrogenic activity in the same *in vivo* assay, even at doses as high as 1,000 mg/kg/day (Sivaraman et al., 2018). Routledge et al. (1998) tested MP, EP, PP, BP as well as pHBA, in the *in vitro* recombinant yeast estrogen screen and determined that these parabens are weakly positive in this assay, with a relative potency of BP > PP > EP > MP, whereas pHBA had no activity. It has to be stressed that in the *in vitro* recombinant yeast estrogen screen assay used by Routledge et al. (1998) the potencies of the parabens were several orders in magnitudes weaker than the endogenous natural hormone 17 β-estradiol, e.g. approximately 2,500,000-fold below for MP. This result also suggests that PP is less biologically active than BP and that using the BP data to fill in data gaps for PP in the final risk assessment, without refinement of potency estimates, will result in a relatively conservative assessment for PP.

### Caffeine and its metabolites case study

In the case of CA and its main metabolites, TP, PX and TB, the assumption was that there was a systemic toxicity data gap for CA and to objective was to determine which of its main metabolites was the most suitable biological analogue which could be used to read across with high confidence. Each of the methylxanthines is able to elicit changes in the expression of a number of genes in exposed cells, as compared to vehicle treated controls, particularly at the highest concentration tested. However, TP and CA are the most active of the group across the cell types evaluated here, with the cardiomyocytes being particularly susceptible to these methylxanthines. Further, the transcriptional profile elicited by both CA and TP in cardiomyocytes shares a high degree of similarity, with 545 genes being up-regulated and 59 being down-regulated by these two methylxanthines. The transcriptional profile of CA and TP has been evaluated in human primary hepatocytes, at various time points and concentrations, as part of the Japanese Toxicogenomics Project consortium (TGP) work (Igarashi et al., 2015), and this data is available for analysis (Open TG-GATEs; available from http://toxico.nibio.go.jp/english/index.html). However, the concentrations for CA and TP tested under the TGP program are higher than the concentrations we have evaluated, and a direct comparison with our data is not possible. However, the analysis of this data comparing the transcriptional response of the primary human hepatocytes to CA (7,500 μM) and TP (10,000 μM) after 8 h of exposure clearly indicates a robust overlap in the response. These similarities further support our conclusion that TP is a biological relevant analogue to read across for CA assessment.

Pathway enrichment analysis of the gene sets identified as being susceptible to modify its expression after exposure of cardiomyocytes to CA and TP, indicate that these methylxanthines have the potential to upregulate the TNFA signaling *via* NFKB pathway, hypoxia and UV responses, as well as various kinases regulated pathways (i.e. p53, KRAS, Pi3K-AKT-MTOR) among others. The cMAP analysis also indicate the potential to modify the PI3K, IKK, MTOR pathways as well as the ATPase activity, mostly acting as inhibitors. Research from multiple authors indicate that methylxanthines act *via* different mechanisms, including antagonism of purinergic P1 receptors, mainly adenosine A1 and A2A receptors (Fredholm et al., 1999). Theophylline has been used in the clinic since 1937 for the treatment of respiratory diseases including asthma and chronic obstructive pulmonary disease (COPD). However, in most treatment guidelines, xanthines have now been consigned to third-line therapy because of their narrow therapeutic window and propensity for drug-drug interactions. However, lower than conventional doses of TP considered to be bronchodilator are now known to have anti-inflammatory actions of relevance to the treatment of respiratory disease. The molecular mechanism(s) of action of TP are not well understood, but several potential targets have been suggested including non-selective inhibition of phosphodiesterases, inhibition of phosphoinositide 3-kinase (PI3K), adenosine receptor antagonism and increased activity of certain histone deacetylases. As indicated, our cMAP analysis clearly shows that the transcriptional profile elicited by each of the four methylxanthines evaluated has high similarity to the one elicited by known PI3K and mTOR inhibitors such as wortmannin, sirolimus, torin1 and 2, among others, as well as with IGF-1 inhibitors such as linsitinib, BMS-754807and BMS-536924 (Figure 4). It has been established that all methylxanthines interact with adenosine receptors, acting as a non-selective antagonist for A_1_, A_2A_, A_2B_ and A_3_ adenosine receptors, however, most of their effects appear to be dependent on the interaction with A_1_ and A_2A_ adenosine receptors (Yasui et al., 2000). However, in our cMAP analysis no connectivity was found with chemicals known to interact with adenosine receptors, even though there are some adenosine receptor antagonist represented in the Clue database, such as CGS-15943, MRS-1334, PSB-1115, and MRS-1220. The cMAP analysis of the genes whose expression was modified by CA or TP in human primary hepatocytes (TG-GATEs) did not result in connections with any of the adenosine receptor antagonist represented in the Clue database either. This apparent discrepancy could be due to differences in potency of these ligands and the methylxanthines, and or the different transcriptome platforms used to generate the profiles in the Clue database (L1000; Lamb et al., 2006) and the ones used in our case (TemO-seq, Biospyder) and the TGP work (Affymetrix, ThermoFisher). Methylxanthines also interact with some phosphodiesterase isoforms (Lazzaroni et al., 1990), and are also known to affect cell growth, proliferation, and energy metabolism by inhibiting PI3K and the mTOR signaling pathways (Zhou et al., 2010; Tariqul Islam et al., 2019). Our pathway enrichment and cMAP analysis support these mechanisms of action for the methylxanthines. The predominant mechanism of action of TP has traditionally been ascribed to non-selective inhibition of phosphodiesterase enzymes (Nicholson and Shahid, 1994), but there is increasing evidence that some of the clinical effects of TP might be due to other mechanisms of action such as increasing histone deacetylase enzyme(s) activity (HDAC; Ito et al., 2002), or interference with certain intracellular kinases (To et al., 2010); the latter mechanism has also been suggested to account for the ability of TP to reverse glucocorticosteroid insensitivity in patients with COPD. TP exhibited an expected concentration dependent antagonism of A1, A2A, A2B and A3 receptors (van Mastbergen et al., 2012), and modest inhibition of phosphodiesterase PDE2, 3, and 10, and particularly on PDE2A1 and only at relatively high concentrations (10^−4^ M) (van Mastbergen et al., 2012). TP has also been shown to inhibit PARP-1 in human pulmonary epithelial cells (Mooren et al., 2005). Theobromine is a phosphodiesterase (PDE) inhibitor and increases intracellular cyclic adenosine monophosphate (cAMP) (Sugimoto et al., 2014). cAMP activates the cAMP‐response element‐binding protein (CREB) which, in turn, induces the expression of specific genes. TB inhibits the Akt-mammalian target of rapamycin mTOR signal in *vitro* as well as in *vivo* (rat) systems (Sugimoto et al., 2014). Mammalian target of rapamycin (mTOR) is a serine/threonine protein kinase that is activated by Akt. Our analysis is also concordant with this activities associated with TB.

Concentrations of TP, TB PX or CA eliciting significant toxicity *in vivo* do not seem to be due to the antagonistic effect on purinergic receptors, but by an unknown mechanism of action causing systemic toxicity. For example, the developmental toxicity determined for TP, TB or PX such as reduction in fetal body weight gain and changes in ossification such as increased occurrence of supernumerary ribs, occurs only at maternally toxic doses (NTP, 1984; Khera, 1985). The main pathways involved in skeletal development are Notch, Hedgehog, FGF, and canonical Wnt pathways (Long, 2011). However, none of these pathways were particularly enriched in our analysis. This could be due to the difference in concentrations we have tested in our *in vitro* approach *versus* the one used *in vivo* or to the unsurmountable differences between the response of cultured cells *versus* an entire organism. One of the limitations that are associated with the methods and approaches that we have used in the current work is related with the cell types we have evaluated. Although we have tried to cover a relatively wide biological space with the four cell types selected for the transcriptional response analysis, by no means have we covered all the potential cell type-specific response to chemical exposure. A larger palette of cell types will have to be evaluated and integrated in the context of individual cell type-response, as well as in the context of the response of the entire set of cell types evaluated, in order to accomplish this goal. A particular limitation of the cell types evaluated here is their limited metabolic capabilities. For a more comprehensive transcriptional response analysis of any given chemical, the transcriptional response of the parent compound as well as its main metabolites must be obtained. In our studies, we have covered this limitation by including in the analysis the main metabolite of the short alkyl parabens, p-Hydroxybenzoic acid, in the first case study, and the main metabolites of caffeine in the second case study. For other chemicals, metabolism of the parent compound must be addressed, either by including a metabolically competent set of cell types (i.e. HepaRG cells, 3D skin models), or by assessing the response to each of its main metabolites. The same must be true when gathering data from other types of assays, such as pharmacological profiling.

In all, the pathway enrichment and cMAP analysis of the CA, TP and TB transcriptional data we have generated in cultured cells clearly indicates that the *in vitro* response is highly similar to the response observed *in vivo* for these methylxanthines, and that the approach of using transcriptional profiling to define relevant biological activity of chemicals being considered for a read across exercise result is a reliable method to determine the most similar biologically active analogue of a particular target chemical.

## Conclusion

The results presented in this study, for both the short alkyl chain parabens and caffeine and its metabolites case studies, indicate that transcriptional profiling derived from exposure of a limited panel of cell types to the chemicals of interest, provides a practical solution to identify the most suitable analog for chemicals of interest based on biological activity similarities for RAX. In the case of the short alkyl chain parabens, the target chemical was PP and the potential analogs to read across were MP, EP and BP. Based on the number of genes whose expression was modified by each paraben in each cell type evaluated, with the MCF-7 cells being to most sensitive to these parabens, the pathways affected and the connectivity to other active chemicals, it can be concluded that the biological activity of PP has the highest similarity the one of BP and thus the data from BP can be used to read across and fill the hypothetical data gaps for PP with high confidence. In the case of CA and its main metabolites TP, PX and TB, CA was selected as the target chemical while its main metabolites were considered as the potential analogs to read across. The results indicate that the most robust transcriptional response was elicited by CA and TP in the four cell types evaluated, with the iCell cardiomyocytes being the most susceptible to these methylxanthines. At gene expression, pathway enrichment and cMAP analysis levels, the highest similarities were identified between CA and TP. Thus, these two methylxanthines are the closest biological analogs, and it can be concluded that the data from TP can be used to read across and fill the hypothetical data gaps for CA with high confidence. Overall, the data demonstrates the utility of a transcriptional profiling of structurally related chemicals to provide one robust stream of data to characterize similarity in biological activity among a given group of chemicals being considered to cover data gaps.

## Data Availability

The complete gene expression data have been deposited in the National Center for Biotechnology Information Gene Expression Omnibus (GEO) (https://www.sciencedirect.com/science/article/pii/S0278691520304294?via%3Dihub Edgar et al., 2002) and are accessible through GEO Series accession number GSE218902.
